# Consumption of Milk Protein or Whey Protein Results in a Similar Increase in Muscle Protein Synthesis in Middle Aged Men

**DOI:** 10.3390/nu7105420

**Published:** 2015-10-21

**Authors:** Cameron J. Mitchell, Robin A. McGregor, Randall F. D’Souza, Eric B. Thorstensen, James F. Markworth, Aaron C. Fanning, Sally D. Poppitt, David Cameron-Smith

**Affiliations:** 1 Liggins Institute, University of Auckland, Auckland 1142, New Zealand; cameron.mitchell@auckland.ac.nz (C.J.M.); r.dsouza@auckland.ac.nz (R.F.D.); e.thorstensen@auckland.ac.nz (E.B.T.); j.markworth@auckland.ac.nz (J.F.M.); 2 School of Biological Sciences, University of Auckland, Auckland 1142, New Zealand; robinmcgregor@gmail.com (R.A.M.); s.poppitt@auckland.ac.nz (S.D.P.); 3 Cardiovascular and Metabolic Disease Center, College of Medicine, Inje University, Busan 614-735, Korea; 4 Fonterra Research and Development Centre, Palmerston North 4442, New Zealand; Aaron.Fanning@fonterra.com

**Keywords:** fractional synthetic rate, amino acids, stable isotopes, milk protein, whey protein, muscle

## Abstract

The differential ability of various milk protein fractions to stimulate muscle protein synthesis (MPS) has been previously described, with whey protein generally considered to be superior to other fractions. However, the relative ability of a whole milk protein to stimulate MPS has not been compared to whey. Sixteen healthy middle-aged males ingested either 20 g of milk protein (*n* = 8) or whey protein (*n* = 8) while undergoing a primed constant infusion of ring ^13^C_6_ phenylalanine. Muscle biopsies were obtained 120 min prior to consumption of the protein and 90 and 210 min afterwards. Resting myofibrillar fractional synthetic rates (FSR) were 0.019% ± 0.009% and 0.021% ± 0.018% h^−1^ in the milk and whey groups respectively. For the first 90 min after protein ingestion the FSR increased (*p* < 0.001) to 0.057% ± 0.018% and 0.052% ± 0.024% h^−1^ in the milk and whey groups respectively with no difference between groups (*p* = 0.810). FSR returned to baseline in both groups between 90 and 210 min after protein ingestion. Despite evidence of increased rate of digestion and leucine availability following the ingestion of whey protein, there was similar activation of MPS in middle-aged men with either 20 g of milk protein or whey protein.

## 1. Introduction

Ageing is associated with a loss of muscle mass, strength, and ultimately physical function; a condition which is termed sarcopenia [1]. The age at which muscle mass and strength begin to decline depend on lifestyle factors such as diet and physical activity, however, the 4th or 5th decade of life is often cited as the starting point for the sarcopenic process [2,3]. Sarcopenia is a major public health concern with over 50 million people currently affected worldwide and this number is projected to increase to 200 million by 2050 [4].

The identification of cost effective and readily available proteins which lead to maximal stimulation of muscle protein synthesis (MPS) in the middle aged population may be important in the development of dietary interventions to maintain muscle mass and prevent or delay sarcopenia. Muscle size is determined by the balance between MPS and muscle protein breakdown (MPB) [5]. In the postabsorptive (fasting) state, MPB exceeds MPS and protein balance becomes negative. When a protein containing meal is consumed, MPB is supressed and MPS is increased such that protein balance becomes positive [6]. In young healthy adults not undergoing resistance training postabsorptive muscle loss is equal to postprandial muscle gain and muscle size is maintained over time. Old age is associated with a decreased MPS response to protein ingestion, termed anabolic resistance [7]. It is difficult to separate the effects of ageing *per se* from the decreased physical activity which accompanies aging; nonetheless, it is likely that age-related anabolic resistance at least partially underlies the progression of sarcopenia [8].

There have been a multitude of research studies which have looked at the ability of different protein sources to stimulate MPS in young men [9,10,11,12] and in older adults [13,14,15], however, there have been very few investigations in middle aged men [16]. Because middle age appears to be the time when the progression towards sarcopenia begins [2] an effective dietary intervention in this population could potentially slow or delay early loss of muscle strength and function.

A number of studies have compared the ability of different milk based and plant based protein fractions to stimulate MPS. It has been shown that the ingestion of fluid milk [12] or the whey protein sub fraction [17] result in greater post exercise MPS and anabolic signaling [18] compared to the same quantity of plant based proteins such as soy. Complete milk protein is composed of 20% whey protein which is rapidly digested and 80% micellar casein which is digested much more slowly [15]. Whey protein ingestion has been shown to result in a greater post exercise MPS response compared to micellar casein [15,19], however, when casein was processed, the results were equivocal [10,14,15]. Some studies have found that in both young and old men calcium caseinate ingestion results in a similar, albeit delayed, MPS response after resistance exercise when compared to whey [10,14] whereas in older men at rest hydrolyzed casein resulted in less MPS than whey [15]. Although whey protein has consistently been shown to robustly stimulate MPS it is produced in limited quantities relative to milk protein concentrates [20] and has different functional properties when incorporated into whole food products [21]. Thus efficacious and varied ingredients are necessary to expand the availability and range of foods which maximally stimulate MPS.

Increases in MPS are dependent on the initiation of protein translation which is primarily regulated by the pathway centred on the mammalian target of rapamycin complex 1 (mTOR) [22]. The mTOR pathway is sensitive to both muscle contraction and nutritional stimuli. The mechanism by which muscle contraction stimulates mTOR is not fully understood, however, recent work has elucidated how amino acids (AA), specifically leucine, activate the mTOR pathway and stimulate protein synthesis [23,24]. Given the primary role of leucine as a nutritional regulator of the mTOR pathway and data showing the superiority of faster digested proteins with higher leucine content in stimulating MPS, multiple authors have proposed that the peak concentration of leucine in the blood may be an important “trigger” to stimulate muscle anabolism [25,26,27].

The purpose of the present investigation was for the first time to compare the MPS responses to complete milk protein and whey protein in middle aged men. The secondary objectives were to assess the effects of these proteins on mTOR pathway activation and to test the hypothesis that peak blood leucine and essential amino acid (EAA) content is the primary determinant of MPS in response to protein feeding.

## 2. Experimental Section

### 2.1. Subjects

Sixteen healthy middle aged men (45–60 years) were recruited to take part in the study through newspaper advertisements; all subjects who commenced the trial completed it. Participants were sedentary to recreationally active had a BMI of less than 30 kg/m^2^, were non-smokers, free of cardiovascular, musculoskeletal, or metabolic conditions and not taking any medication. Subject characteristics are shown in Table 1. Baseline blood biochemistry analysis was performed using a Hitachi 902 autoanalyzer, (Hitachi High Technologies Corporation, Tokyo, Japan) by enzymatic colorimetric assay (Roche, Mannheim, Germany). The homeostatic model assessment (HOMA) insulin resistance was calculated based on the standard equation [28]. Before commencement of the study the protocol was explained to the participants and written consent was obtained. Ethics approval was provided by the Auckland District Health Board Research Review Committee.

**Table 1:** Beverage composition. * Total protein calculated as total nitrogen × 6.25.

	Milk Protein Concentrate	Whey Protein Concentrate
Energy (kJ)	397	412
Protein * (g)	20	20
Carbohydrate (lactose) (g)	0.34	1.79
Fat (g)	1.42	1.16
Aspartic acid + Asparagine (g)	1.63	2.19
Threonine (g)	0.94	1.61
Serine (g)	1.17	1.14
Glutamic acid + Glutamine (g)	4.85	4.02
Proline (g)	2.10	1.32
Glycine (g)	0.40	0.39
Alanine (g)	0.73	1.19
Valine (g)	1.29	1.21
Isoleucine (g)	1.11	1.36
Leucine (g)	2.09	2.25
Tyrosine (g)	1.11	0.61
Phenylalanine (g)	1.06	0.69
Lysine (g)	1.73	1.91
Histidine (g)	0.58	0.35
Arginine (g)	0.75	0.55
Cystine (g)	0.16	0.53
Methionine (g)	0.61	0.53
Tryptophan (g)	0.35	0.42

### 2.2. Experimental Protocol

At least one week prior to the experimental trial subjects underwent a dual energy x-ray absorptiometry (DXA, Lunar Prodigy, GE, Waltham, MA, USA) scan to quantify lean, fat mass, and total mass. The results obtained from the DXA scan were then used to estimate the participants’ energy requirements using the Harris-Benedict equation [29] and calculate the isotope infusion rate. Participants were asked to refrain from intense physical activity for two days prior to the experimental trial and were given a standard evening meal (30% fat, 55% carbohydrate, 15% protein) containing a third of their estimated daily energy requirements. The participants consumed the meal prior to 22:00 and then consumed nothing except water for the rest of the evening. Participants arrived fasted to the lab at 07:00, a 20-gauge plastic catheter was inserted into an antecubital vein and a baseline blood sample was obtained. A slow saline drip was used to keep the catheter patent and a heating blanket was used to arterialize the blood samples. A muscle biopsy (100 mg) was obtained from the vastus lateralis muscle with a Bergström needle modified for manual suction under local anesthesia (2% xylocaine). Visible fat and connective tissue was dissected away from the biopsy and it was immediately frozen in liquid nitrogen.

After the fasting biopsy a second cannula (22-gauge) was inserted into a contralateral antecubital vein and a primed constant infusion of l-[ring-^13^C_6_] phenylalanine (prime: 2 μmol·kg^−1^; infusion: 0.05 μmol·kg^−1^·min^−1^) was commenced and maintained for the remainder of the trial. After two hours of rest, a second biopsy was obtained from the contralateral leg. Participants then consumed one of two study beverages within 5 min. Blood samples were obtained 15, 30, 45, 60, 75, 90, 120, 180, and 210 min after the beverage was consumed. Additional biopsies were obtained at 90 min and 210 min after ingestion of the drink.

### 2.3. Study Beverages

Participants were randomly assigned to consume either 20 g of Milk Protein Concentrate (MPC) 485 (Fonterra Co-operative Group Ltd., Auckland, New Zealand) or 20 g of Whey Protein Concentrate (WPC) 392 (Fonterra Co-operative Group Ltd., Auckland, New Zealand) in a double blind fashion. Both beverages were supplied by Fonterra Co-operative Group Ltd. Each ingredient was dissolved in 350 mL of water and contained less than 2 g of fat and less than 2 g of carbohydrate (Table 2). Free l-[ring-^13^C_6_] phenylalanine was not added to either beverage because it was anticipated that each beverage would have different rates of phenylalanine appearance thus the addition of tracer might cause an increase in tracer to trace ratio in the MPC group.

**Table 2:** Subject Characteristics. Baseline subject characteristics in the fasted state. HOMA, Homeostasis Model Assessment; LDL-C, low density lipoprotein; HDL, high density lipoprotein. *P* values are the result of independent sample *t*-tests. Means ± standard deviations.

	WPC (*n* = 8)	MPC (*n* = 8)	*P*
Age (year)	52.6 ± 3.9	52.1 ± 6.4	0.853
Body mass (kg)	88.6 ± 13.4	74.5 ± 6.1	0.024 *
Body fat (%)	24.3 ± 9.2	19.2 ± 7.4	0.243
Glucose (mmol/L)	5.51 ± 0.41	5.46 ± 0.36	0.800
Insulin (μU/mL)	8.47 ± 4.39	7.46 ± 3.41	0.616
HOMA insulin sensitivity	2.11 ± 1.19	1.81 ± 0.80	0.565
Triglycerides (mmol/L)	1.42 ± 0.58	1.31 ± 0.42	0.671
LDL (mmol/L)	3.60 ± 0.97	3.11 ± 1.16	0.376
HDL (mmol/L)	1.26 ± 0.25	1.25 ± 0.30	0.943
Total cholesterol (mmol/L)	5.86 ± 1.06	5.26 ± 1.31	0.293

### 2.4. Fractional Synthetic Rate

Muscle samples (40–50 mg) were homogenized for 40 s at 20 Hz using a TissueLyser (Qiagen, Venlo, Netherlands) in buffer (10 μL/mg 25 mM Tris 0.5% *v*/*v* Triton X-100) and protease/phosphatase inhibitor cocktail (HaltTM Protease and Phosphatase Inhibitor Cocktail, Thermo Scientific, cat. 78442). Samples were then centrifuged at 15,000 *g* for 10 min at 4 °C. The supernatant was collected for western blot analysis and the solid pellet was processed as previously described to calculate the rate of protein synthesis for a myofibrillar enriched fraction [30]. The myofibrillar pellet was hydrolyzed in 6 M HCL at 110 °C overnight. The free AA were then purified using ion-exchange chromatography and converted to their *N*-acetyl-*N*-propyl ester derivatives for analysis by using gas chromatography-combustion-isotope ratio mass spectrometry (IRMS model: Delta Plus XP; Thermo Finnagan) [31]. A standard was run every 10 injections. Fractional synthetic rate was calculated using the equation:

(1) $$FSR\left(\%h^{-1}\right)=\left[\frac{E_{m2}-E_{m1}}{E_{plasma}\;\cdot \;t}\right]100$$

where, FSR is the fractional synthetic rate, *E_m2_* and *E_m1_* are the protein bound enrichments from the myofibrillar enriched fraction of the muscle biopsies and thus their difference is the change in bound protein enrichment between two time points; *E_plasma_* is the time weighted mean phenylalanine enrichment from the blood samples taken during the incorporation period; and *t* is the tracer incorporation time. Plasma phenylalanine enrichments were used as the precursor pool, this method results in slightly lower FSR values compared to using the intramuscular free amino acid pool. Plasma ^13^C_6_-phenylalanine was measured using established methods [32,33]. Briefly, the isotopic enrichment of plasma samples was determined by negative chemical ionization gas chromatography–mass spectrometric analysis of a heptafluorobutyric, *n*-propyl derivative. ^13^C_6_-phenylalanine enrichment was measured using methane negative chemical ionization GC-MS (Agilent 5973 EI/CI MSD with an Agilent 6890 GC). A Phenomenex ZB-1MS capillary column was used to separate the derivative of phenylalanine. Selected ion chromatograms were obtained by monitoring ions *m/z* 383 and 389 for l-phenylalanine and l-[^13^C_6_] phenylalanine, respectively. Isotope enrichment in mole % excess was calculated from peak area ratios at isotopic steady state and baseline. The final value for all determinations was corrected using an enrichment calibration curve. All mass spectrometry analyses were performed by Metabolic Solutions Inc. (Nashua, NH, USA).

### 2.5. Western Blotting

Total protein content of the muscle homogenate supernatant (described above) was determined using a BCA-protein kit as per the manufacturer’s instructions (Thermo Fisher Scientific, Waltham, MA, USA). Aliquots of 20 µg total protein were prepared, suspended in Laemmli buffer, boiled, and subjected to SDS-PAGE. The four muscle biopsy samples collected from each participant were always run in contiguous lanes on the same gel. Proteins were transferred to a PVDF membrane, using the Trans-Blot^®^ Turbo™ Transfer System (Bio-Rad, Hongkong, China) and blocked in 5% BSA/Tris Buffer Saline/0.1% Tween 20 (TBST) for 2 h at room temperature, followed by overnight incubation at 4 °C with gentle agitation with primary antibodies (1:1000) (p-P70S6K (Thr421/Ser424) (Cell Signalling, 9204S), p-Akt (Ser473) (Cell Signalling, 4056S), p-rps6 (Ser235/236) (Cell Signalling, 4865S), p-rps6 (Ser240/244) (Cell Signalling, 2215S), and GAPDH (Abcam, ab9485)). The following morning the membranes were washed for 30 min with TBST and probed with HRP conjugated goat anti-rabbit or goat anti-mouse secondary antibodies (Jackson ImmunoResearch, West Grove, PA, USA) for 1 h at room temperature. Following 30 min further washing in TBST, antibody binding was visualized using ECL Select Western blotting detection reagent (Amersham, UK) and chemiluminescent signals were captured using a ChemiDoc™ MP Imaging System (Bio-Rad, Hongkong, China). Densitometry analysis of protein bands was performed using Image J software. Abundance of proteins of interest was normalized for protein loading by stripping and re-probing membranes for GAPDH.

### 2.6. Plasma Amino Acid Concentrations

Samples of plasma for measurement of AA were deproteinized by tungstate precipitation. An aliquot of sample (20 µL) was mixed with 160 µL 0.04 M H_2_SO_4_ (containing 15 µM norvaline, the internal standard) and left to stand on ice for 2 min, 10% sodium tungstate (Na_2_WO_4_) was then added (20 µL) and the solution vortex mixed. The mixture was then centrifuged at 4 °C and 14,000 *g* for 10 min.

Plasma AA concentrations in the resulting supernatant were then measured by a fluorescent derivitization utilizing the reaction of aminonitrogen with 6-aminoquinolyl-*N*-hydroxysuccinimidyl carbonate and subsequent separation by ultra-high pressure liquid chromatography (UHPLC). Standards and quality control samples were prepared and analyzed similarly.

The UHPLC system consisted of a Thermo Scientific Dionex Ultimate 3000 pump, autosampler (maintained at 10 °C), column oven and fluorescence detector (set at Ex 250 nm, Em 395 nm) (Thermo Scientific, Dornierstrasse, Germany), and a Kinetex 1.7 µm C18 100A 100 × 2.1 mm column, preceded by a Krudkatcher inline filter (Phenomenex, Auckland, New Zealand) at 45 °C.

The mobile phase was a buffer, (80 mM sodium acetate, 3 mM triethylamine, 2.67 µM disodium calcium ethylenediaminetetraacetic acid) at pH 6.43, run with a complex gradient of acetonitrile from 2% to 17% (balance, water) over 24 min.

Data were captured directly by computer with Chromeleon 7.1 software (Thermo Scientific). AA concentrations in the samples were calculated from standard curves generated for each AA from the standard injections.

### 2.7. Statistical Analysis

Baseline differences in subject characteristics were assessed with *t* tests. Differences in protein synthesis, plasma AA concentration, and anabolic signaling were assessed with two-way repeated measures ANOVA with time as within subject factor and group as a between subject factor. Between group differences were assessed with Sidak’s *post hoc* method. *A priori* sample size calculations were conducted using a power of 80% based the variance and difference in MPS observed between whey and casein at rest in young men [19]. All data are reported as mean ± standard deviation (SD) in the text and tables and mean ± standard error (SE) in the figures. Significance is set at an alpha of less than or equal to 0.05.

## 3. Results

### 3.1. Subjects

Subject characteristics are shown in Table 2. There were no differences in any anthropometric characteristics or biochemistry at baseline.

### 3.2. Plasma Amino Acids

**Figure 1:**
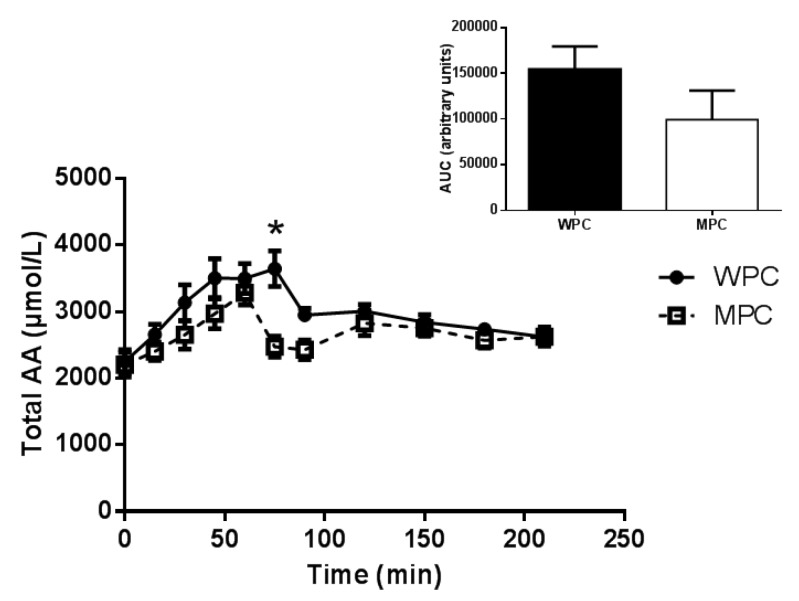
Plasma Total Amino Acids (AAs). Time course of plasma total AA concentration following the ingestion of 20 g of MPC or WPC (main panel). Solid line and filled circles resent WPC; dashed line and open squares represent MPC. The insert shows the area under the curve (AUC) for 210 min following the ingestion of the protein beverage. *, different from MPC at the same time point *p* ≤ 0.05. Error bars represent standard error of the mean.

The concentration of total plasma amino acids is displayed in Figure 1. A significant group × time interaction (*p* < 0.05) was apparent such that total AA concentration was greater in the WPC group compared with the MPC group at 75 min post beverage consumption (Figure 1). Total plasma AA area under the curve (AUC) was not different after the consumption of MPC or WPC (*p* = 0.206).

**Figure 2:**
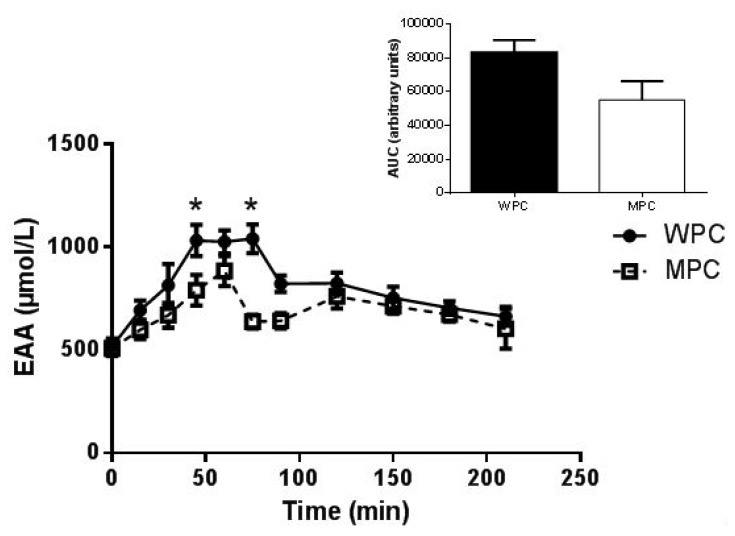
Plasma Essential Amino Acids (EAAs) excluding Leucine. Time course of plasma EAA excluding leucine concentration following the ingestion of 20 g of MPC or WPC (main panel). Solid line and filled circles resent WPC; dashed line and open squares represent MPC. The insert shows the area under the curve (AUC) for 210 min following the ingestion of the protein beverage. *, different from MPC at the same time point *p* ≤ 0.05. Error bars represent standard error of the mean.

There was significant group × time interaction (*p* < 0.05) for plasma EAA excluding leucine and post hoc analysis revealed that EAA concentration was greater in the WPC group compared to the MPC group 45 and 75 min post beverage consumption. The AUC for EAAs excluding leucine was also overall higher following WPC ingestion compared to MPC however this trend did not reach statistical significance (*p* = 0.077, Figure 2).

**Figure 3:**
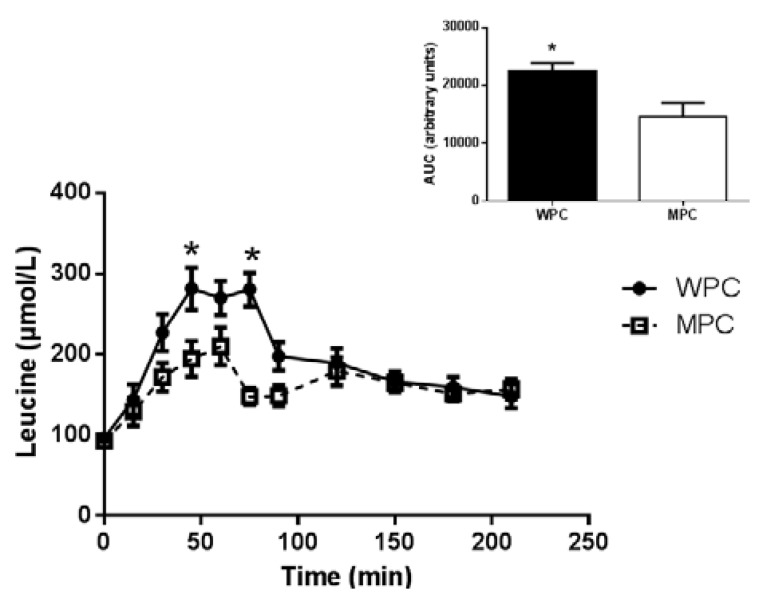
Plasma leucine. Time course of plasma leucine concentration following the ingestion of 20 g of MPC or WPC (main panel). Solid line and filled circles resent WPC; dashed line and open squares represent MPC. The insert shows the area under the curve (AUC) for 210 min following the ingestion of the protein beverage. *, different from MPC at the same time point *p* ≤ 0.05. Error bars represent standard error of the mean.

There was significant group × time interaction (*p* < 0.05) and post hoc analysis revealed that plasma leucine concentration was greater at 45 and 75 min following WPC ingestion compared to MPC. The AUC of leucine was also greater following WPC consumption compared to MPC protein consumption (*p* = 0.012, Figure 3).

**Figure 4:**
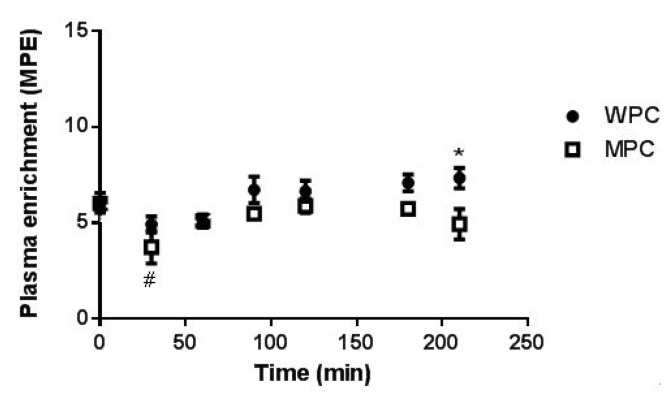
Plasma ^13^C_6_ enrichment. The tracer enrichment of ^13^C_6_ phenylalanine over the infusion period is shown. The closed circles represent WPC and the open square represent MPC. *, different from MPC at the same time point *p* ≤ 0.05; #, main effect for difference from 0 time point *p* ≤ 0.05. Error bars represent standard error of the mean.

The plasma phenylalanine enrichment is shown is Figure 4. There were no group differences in plasma enrichment ratio with the exception of a slightly greater enrichment in the WPC group compared to the MPC group at 120 and 210 min after beverage consumption (*p* < 0.05). There was a main effect for a decrease in plasma enrichment at 30 min after beverage consumption. Plasma phenylalanine rate of appearance (Ra) increased for 60 min following beverage consumption and decreased only in the WPC at 180 and 210 min following beverage consumption (supplementary material).

### 3.3. Anabolic Signaling

**Figure 5:**
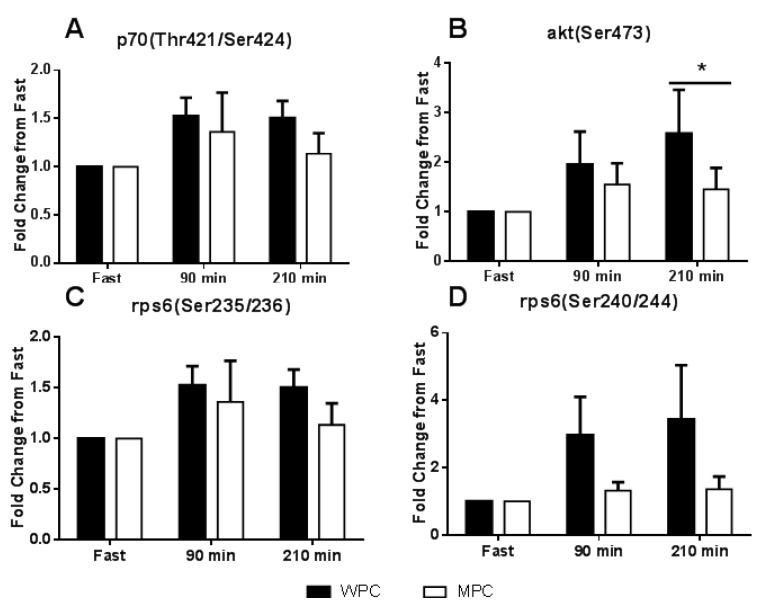
Muscle anabolic signaling. (**A**) Change in p70S6K phosphorylation at threonine 421 and serine 424 sites from the fasted state, solid bar represent WPC ingestion and open bars represent MPC ingestion. (**B**) Change in Akt phosphorylation at the serine 473 site from the fasted state. (**C**) Change in ribosomal protein S6 (rpS6) phosphorylation at the serine 235/236 sites from the fasted state. (**D**) Change in ribosomal protein S6 (rpS6) phosphorylation at the serine 240/244 sites from the fasted state. The solid horizontal line represents a main effect for time. *, different from fast *p* ≤ 0.05. Error bars represent standard error of the mean.

P70S6K phosphorylation on Thr421/Ser424 did not change over time (*p* = 0.182) and was not different between groups (*p* = 0.368, Figure 5a). There was a main effect for increased Akt phosphorylation (*p* = 0.044) on Ser473 at 90 min following the consumption of the protein beverages, but there were no differences between groups (*p* = 0.374, Figure 5b). Rps6 phosphorylation was assessed on both the Ser235/236 and Ser240/244 residues which did not change over time (Ser235/236: *p* = 0.108, Ser240/244: *p* = 0.096) and were not different between groups (Ser235/236: *p* = 0.679, Ser240/244: *p* = 0.266, Figure 5b,c). Sample western blots are shown in supplementary Figure S1.

### 3.4. Muscle Fractional Synthetic Rate

**Figure 6:**
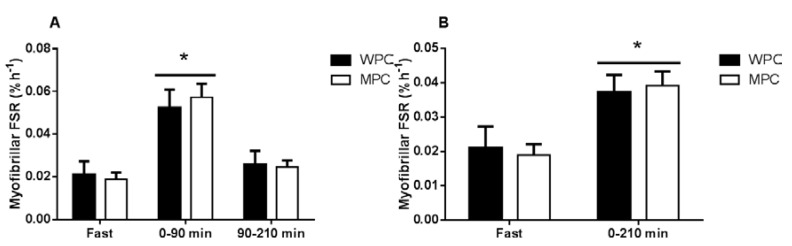
Muscle fractional synthetic rate (FSR). A) The course of myofibrillar protein synthesis following the ingestion of 20 of MPC (open bars) or WPC (black bars). B) The aggregate myofibrillar protein synthesis following the ingestion of 20 of MPC (open bars) or WPC (black bars). The solid horizontal line represents a main effect for time. *, different from fast *p* ≤ 0.05. Error bars represent standard error of the mean.

Resting muscle FSR was calculated over a 2 h baseline incorporation period and was not different between groups (*p* = 0.991 Figure 6a). The FSR for the first 90 min after the ingestion of the protein beverage was elevated above baseline in both groups (*p* < 0.0001) with no difference between groups (*p* = 0.915). The FSR between 90 min and 210 min after the consumption of the protein beverage was no longer elevated above baseline (*p* = 0.630) and was not different between conditions (*p* = 0.998). When the FSR values are expressed as an aggregate rate from the time of consumption of the protein beverage until 210 min FSR was elevated above baseline (*p* = 0.006) with no difference between groups (*p* = 0.649, Figure 6b). Muscle myofibrillar protein bound enrichments increased in each subsequent biopsy with no differences between groups (supplementary material).

## 4. Discussion

This is the first study to directly compare the effect of total milk protein concentrate and the more well described whey protein on human skeletal muscle FSR. The primary novel finding from this study is that 20 g of protein from MPC was equally as effective as 20 g of protein from WPC at increasing MPS in healthy middle aged men. Many studies have shown the beneficial effects of whey protein on MPS, with the present study providing the additional evidence of efficacy under resting conditions in middle-aged individuals. However, whey comprises approximately 20% of the bovine milk fraction. Thus, whey supply is limited and cost of manufacture is greater than that of the whole milk protein [20]. Therefore, a consideration of the current study is analysis of equivalence in protein synthesis functionality. Previous studies have demonstrated the importance of peak plasma EAA or leucine concentrations following ingestion as the primary determinate of MPS response [25,27]. However, the findings of the current study demonstrate that despite eliciting a significantly lower EAA, and leucine concentration in plasma, milk protein concentrate ingestion resulted in equally robust stimulation of MPS.

Although this is the first MPS study to directly compare milk protein to whey protein in any population, there have been many reports comparing different milk protein fractions to one another as well as to plant based proteins (e.g., soy). Milk based proteins have consistently been shown to result in greater muscle protein synthetic and anabolic signaling response when compared to plant based protein [13,18,19]. A greater content of EAAs, particularly leucine in milk protein is generally cited to explain the superiority of milk proteins [34] because leucine is known to provide an important signal to stimulate anabolic signaling and MPS [35].

Whey protein comprises 20% of milk protein and its ingestion is characterized by a rapid aminoacidemia in the blood and muscle [19]. Casein in its micellar form make up the remaining 80% of milk protein and is characterized by a slow and sustained release of AAs into the blood and muscle [19]. When directly compared to micellar casein, whey has been shown to promote a greater MPS response in both younger [19] and older men [15,36]. In trials where casein has been processed to increase the rate of AA release by forming a hydrolysate or a caseinate the results have been found to be equivocal. Ingestion of casein hydrolysate at rest resulted in a lower MPS response compared to whey [15], but during exercise recovery ingestion of either calcium caseinate or whey resulted in a similar MPS response [10,14]. Trials which have manipulated the rate of aminoacidemia after exercise by providing whey protein as either a 20 g bolus or an equivalent dose over three hours have also shown a larger MPS response to bolus ingestion eliciting more rapid plasma aminoacidemia, suggesting the peak AA concentration may be more important than AA AUC for stimulating MPS [37,38]. However, the rate of aminoacidemia may be less important when muscle is in the rested state. Recent work in both young [39] and older men [40] has shown that in the resting state a rapid *vs*. sustained EAA delivery profile has no effect of MPS. Taken together this suggests that the 2 g of leucine in beverage was sufficient to maximally stimulate MPS at rest but that differences in AA delivery profiles might have caused a divergent MPS response had exercise sensitized the muscle prior to feeding.

The apparent importance of protein source, leucine content, and rate of plasma aminoacidemia has led to the development of the “leucine threshold” model which states that the magnitude of the MPS response will be proportional to the peak concentration of leucine in the plasma [25]. Milk protein contains far more casein than whey and, as expected [41], we show that milk protein ingestion results in a relatively attenuated AA (particularly leucine) response, similar to what is observed with micellar casein. Based on this, it was our hypothesis that MPC would result in a lower MPS response compared to WPC. However, despite marked differences in the plasma EAA and leucine response, we observed equivalent stimulation of MPS in response to either MPC or WPC. It is plausible that in middle aged men at rest the leucine concentration required to maximize MPS is relatively low or that total delivery is more important than peak concentration. It is also conceivable that because of the slower digestion of the MPC a longer incorporation period would be necessary to capture the full MPS response, beyond the 210 min post-ingestion analyzed in the current study. Experimentally, this is unlikely as the MPS response peaked between 0–90 min after nutrition and declined between 90 and 210 min post-nutrition. Thus differences beyond 210 min are likely to be quantitatively small. A number of studies have shown a divergence between plasma leucine concentration and MPS suggesting the leucine threshold model may be incomplete [9,11,42,43].

Middle age is considered to be the period when the age related decline in muscle size, quality, and function begin [2]. This makes middle age adults the ideal target group for interventions which might prevent or delay sarcopenia. Whilst surprisingly few studies have been conducted in middle aged men, in young and men at rest 20 g of whey protein is sufficient to maximize the MPS response to feeding [44,45]. In older men one study has shown that 20 g of whey is sufficient to maximize MPS [44] while another has shown at least 35 g is required [46]. A dose response of MPS in response to protein feeding has not been conducted in middle aged men but based on the studies available it was thought that 20 g of dairy protein would produce a robust if not maximal MPS response in middle aged men. It is plausible that the peak leucine concentration which occurred after the ingestion of the MPC was sufficient to maximize MPS at rest in middle aged men and that the greater leucine concentration achieved following WPC ingestion provide no additional benefit. A divergent MPS response between MPC and WPC might have been observed had a lower dose been given or had an exercise stimulus been used to sensitise the muscle to protein feeding [9,47].

In addition to measurement of MPS, we also characterized the response of members of the mTOR pathway to protein ingestion. There were no differences in phosphorylation status from baseline in any of the measured signaling proteins with the exception of an increased p-Akt (Ser473) at 210 min after the consumption of both protein beverages. Protein ingestion is known to result in a relatively transient increase in anabolic signaling [48]. It may be that 90 min after the consumption of the protein beverage was too late to detect the typical transient rise in signaling kinase phosphorylation. The response of mTOR pathway components to feeding is notably variable between studies even when the same proteins are used [9,42,49,50]. In the current study, a 90 min post feeding biopsy time point was chosen for the calculation of FSR and which may have been suboptimal for the detection of the transient signaling response to feeding. It is likely that, had the subjects performed exercise prior to the ingestion of the protein beverages, there would have been an increased sensitization to feeding and larger anabolic signaling and MPS responses observed [6,50]. It has previously been shown that more divergent MPS and anabolic signaling responses are observed in response to ingestion of a given protein source when combined with an exercise stimulus [42]. Therefore, it is tenable that the inclusion of an exercise stimulus would have resulted in a superior anabolic response to one of the protein sources tested in the current study. Nevertheless, in the resting state, it is clear that no differences in the anabolic signaling or MPS were apparent between MPC and WPC provided at the same dose.

The present study included a measurement of MPS in the rested fasted state. The purpose of this measurement was to show an increase in MPS following feeding, the resting fasted myofibrillar MPS values observed are in line with those reported in the literature albeit slightly lower because of the use of plasma as the precursor pool [37,51]. The two hour incorporation period used to calculate fasted MPS is shorter than the three hour incorporation period often used but has been previously validated [52] and previously used with similar results [53]. The resting fasted measurement does not affect the main conclusion of the paper that MPC and WPC result in similar increases in MPS it simply confirms that in agreement with an extensive body of literature that 20 g of dairy protein increase MPS above resting levels [13,15,19].

Based on the results of the present study, both MPC and WPC can be recommended as an effective strategy to increase MPS in middle age these findings likely translate to both younger and older adults. More work is required to identify the maximal effective dose of these proteins in middle age and older adults. Additional research will also be required before MPC can be recommended as equivalent to WPC after exercise.

## 5. Conclusions

Dairy protein represents a source of high quality protein which has repeatedly been shown to stimulate MPS in both young [45] and older adults [13]. Whey protein has been previously revealed as superior at stimulating MPS, both at rest and after resistance exercise, when compared to micellar casein [19]. However, this is the first study to directly compare WPC to MPC. We show that in middle aged men MPC is equally as effective as WPC as stimulating MPS in the resting state. These findings show that the consumption of milk protein is a simple and cost effective strategy to stimulate MPS in middle aged men.

## Supplementary Materials

Supplementary materials can be accessed at: http://www.mdpi.com/2072-6643/7/10/5420/s1.
