# Supplementary material for: Consumption of Milk Protein or Whey Protein Results in a Similar Increase in Muscle Protein Synthesis in Middle Aged Men
Source: Nutrients. 2015 Oct 21;7(10):8685–99. doi: 10.3390/nu7105420 (PMC4632440; doi:10.3390/nu7105420)
Supplement: Supplementary file 1 [file nutrients-07-05420-s001.docx]

**Supplementary Information**


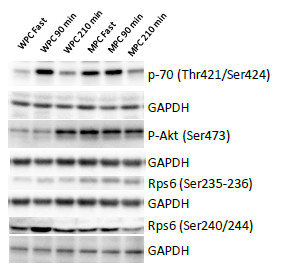


**Figure S1**. Representative western blots.


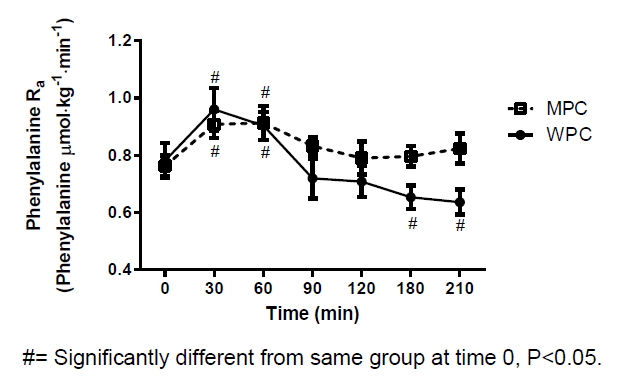


**Figure S2**. Phenylalanine rate of appearance.


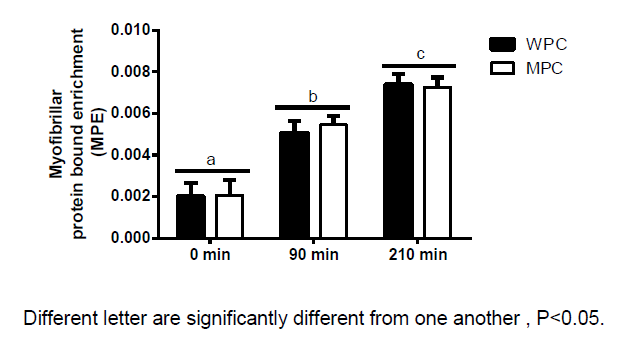


**Figure S3.** Muscle myofibrillar protein bound enrichment.

© 2015 by the authors; licensee MDPI, Basel, Switzerland. This article is an open access article distributed under the terms and conditions of the Creative Commons by Attribution (CC-BY) license (http://creativecommons.org/licenses/by/4.0/).
